# Teaching breaking bad news in a gyneco-oncological setting: a feasibility study implementing the SPIKES framework for undergraduate medical students

**DOI:** 10.1186/s12909-024-05096-9

**Published:** 2024-02-12

**Authors:** Cosima Zemlin, Nasenien Nourkami-Tutdibi, Pascal Schwarz, Gudrun Wagenpfeil, Sybelle Goedicke-Fritz

**Affiliations:** 1 Department of Gynecology and Obstetrics, Faculty of Medicine, Saarland University, Homburg, Germany; 2 Department of General Pediatrics and Neonatology, Saarland University, Homburg, Germany; 3 Institute of Medical Biometry, Epidemiology and Medical Informatics, Saarland University, Homburg, Germany

**Keywords:** Breaking Bad News, SPIKES protocol, Communication skills, Oncology, Teaching, Feasibility study, Role-play, Health communication, Formative self- and peer-assessment, 360°feedback

## Abstract

**Background:**

It is a crucial task for physicians to deliver life threatening information to patients (breaking bad news; BBN). Many aspects influence these conversations on both sides, patients, and doctors. BBN affects the patient-physician relationship, patients’ outcome, and physicians’ health. Many physicians are still untrained for this multi-facetted task and feel unprepared and overburdened when facing situations of BBN. Therefore, any faculties should aim to integrate communication skills into their medical curricula as early as possible. The SPIKES protocol is an effective framework to deliver BBN. Aim of this study is to evaluate the feasibility and obstacles of a BBN seminar and its acceptance and learning curve among undergraduate medical students.

**Methods:**

158 2nd year undergraduate medical students attended a compulsory BBN seminar. The task was to deliver a cancer diagnosis to the patient within a patient - physician role-play in a gyneco-oncological setting before and after a presentation of the SPIKES protocol by the lecturer. The students evaluated important communication skills during these role-plays respectively. Self-assessment questionnaires were obtained at the beginning and end of the seminar.

**Results:**

Most students indicated that their confidence in BBN improved after the seminar (*p* < 0.001). They like the topic BBN to be part of lectures (76%) and electives (90%). Communication skills improved. Lecturer and seminar were positively evaluated (4.57/5).

**Conclusion:**

The seminar significantly increased confidence and self-awareness in delivering life-threatening news to patients among undergraduate medical students. Important learning aspects of BBN and communication skills could be delivered successfully to the participants within a short time at low costs. The integration of communication skills should be implemented longitudinally into medical curricula starting before clinical education to increase the awareness of the importance of communication skills, to decrease anxiety, stress, and workload for future doctors and– most importantly– to the benefit of our patients.

**Supplementary Information:**

The online version contains supplementary material available at 10.1186/s12909-024-05096-9.

## Introduction

Patient - physician conversation is an essential task for daily clinical practice [1–5]. Physicians not only act as communicators to transmit medical information to patients and their relatives, but also as specialists in the disease and as companions and therapists of the patients. Delivering life threatening diagnosis and/or findings negatively influencing a patient’s outcome is described as “breaking bad news” (BBN) in literature and are demanding situations for patients, their relatives, and all members of the medical team [1, 2, 6]. How information is conveyed to a patient affects the patient– physician relationship, the understanding, compliance and can impact the treatment course and response [7]. BBN remains the most challenging aspect within patient-physician relationship [6].

Age, sex, living conditions, medical history and current state of health, socio-cultural background, religious beliefs, philosophy of life, and level of education are important aspects to be considered in patient– physician relationship and communication on both sides [8]. In addition, doctors are also influenced by their beliefs about “what is best for the patient” and may feel powerless and helpless when therapies no longer work, and when they are confronted in revealing this information to their patients [9, 10]. BBN is challenging as bad news should be delivered standardized yet individualized according to each patient´s needs at the same time [1, 2, 6]. To be able to respond to the various needs of the different patients, and to avoid being influenced by own personal perspectives it is of utmost importance to first listen to the patient’s needs and knowledge before the doctor delivers the message that threatens the patient’s existence [11, 12]. Unprepared and untrained physicians and members of the treatment team often feel overburdened and overstressed when facing situations of BBN [9]. They acknowledge that insufficient training in communication and management skills is a major factor that leads to stress, lack of job satisfaction and emotional burnout [7]. In addition they are often unaware of the great impact that manner, mode and setting of these sensitive conversations may have on their patient’s perception, acceptance and compliance [9]. Thus, the way how the patient is informed affects both patients and physicians [7, 13].

The latter is of extreme importance especially in oncologic settings as BBN is always a vulnerable and important moment for the patient and the treating physician. BBN in oncology can mean, to inform the patient about a cancer diagnosis, recurrence or progression of disease, treatment failure, the occurrence of severe side effects, medical malpractice, and other undesirable conditions [3, 11, 14].

In addition, BBN and how sensitive information is conveyed in a healthcare system impacts on working atmosphere and can either create extreme stress on the health care team members or ease working and daily patient care [1, 4, 15, 16]. Delivering bad news is not restricted to oncology though [17]. Delivering bad news is an important competence in most medical subspecialties, such as obstetrics, cardiology, emergency room and many others specialties [5].

The inclusion of BBN competence as a mandatory component of treating critically ill patients in study protocols and guidelines was an important achievement [18]. The SPIKES protocol is a well-evaluated guideline and effective approach to deliver sensitive information to the patient [1]. The protocol describes six steps to be applied while breaking the bad news. The six steps comprise “Setting, Perception, Invitation, Knowledge, Emotions and Strategy and Summary” (SPIKES) [5]. The SPIKES protocol is of great international importance and is also used in Germany as a teaching protocol [5]. According to the SPIKES protocol, the goals of the informed consent dialogue are to gather information from the patient, provide medical information, offer support to the patient, while developing a plan with the patient at the same time. Other approaches/protocols to deliver bad news are e.g., PENS, BREAKS; ABCDE or EPICES [19].

Professional education programs to train communication skills, with a focus on conveying breaking bad news are vital for a trustable patient-physician relationship [3, 4, 11, 12, 15, 16, 20, 21]. Therefore, according to the current master plan for medical studies in Germany, it must be part of the curriculum at medical faculties [22, 23].

Given the importance of communication skill training (CST), more and more medical faculties start to implement longitudinal communication curricula that address this basic skill at multiple stages of the medical training. This allows students a harmonized progress in medical knowledge and CST [24–27]. Throughout Germany, many medical faculties successfully implemented CST into their core curricula [28]. In France e.g., Bonnaud-Antignac used a videotaped simulated interview with actors as an approach to teach students BBN [29]. Before and after participating in an interactive lecture, based on the SPIKES protocol, the students conducted role-plays in between themselves. In our study the group was divided into “actors” (respectively “doctors” and “patients”) and observers. For the evaluation, we used questionnaires based on Likert scales, which dealt with the students’ self-assessment during the course of the seminar (QA), the communication skills of the “doctor” during the respective role-plays (QB), and the evaluation of the seminar (QE) (Supplement 2).

Aim of this study is to evaluate the feasibility and obstacles of a BBN seminar and its acceptance and learning curve among undergraduate medical students.

## Methods

The BBN-seminar was conducted at the Saarland University Medical Center in Homburg/Saar (UKS) as part of the EKM curriculum “Introduction to clinical medicine” (***E****inführung in die****k****linische****M****edizin*) in the 2nd undergraduate year. It is a mandatory seminar in the afternoon. Clinicians are supposed to familiarize pre-clinical students in 90 min with a topic from the clinical routine. A lecturer experienced in BBN, who was trained to conduct the training, led all sessions.

158 2nd year medical students took part in the course in 8 groups with 10–28 participants, respectively. Prior to this seminar, students had not participated in communication skill training. As part of their studies, they had completed an 8-week nursing internship.

### BBN-seminar and evaluation

**Fig. 1:**
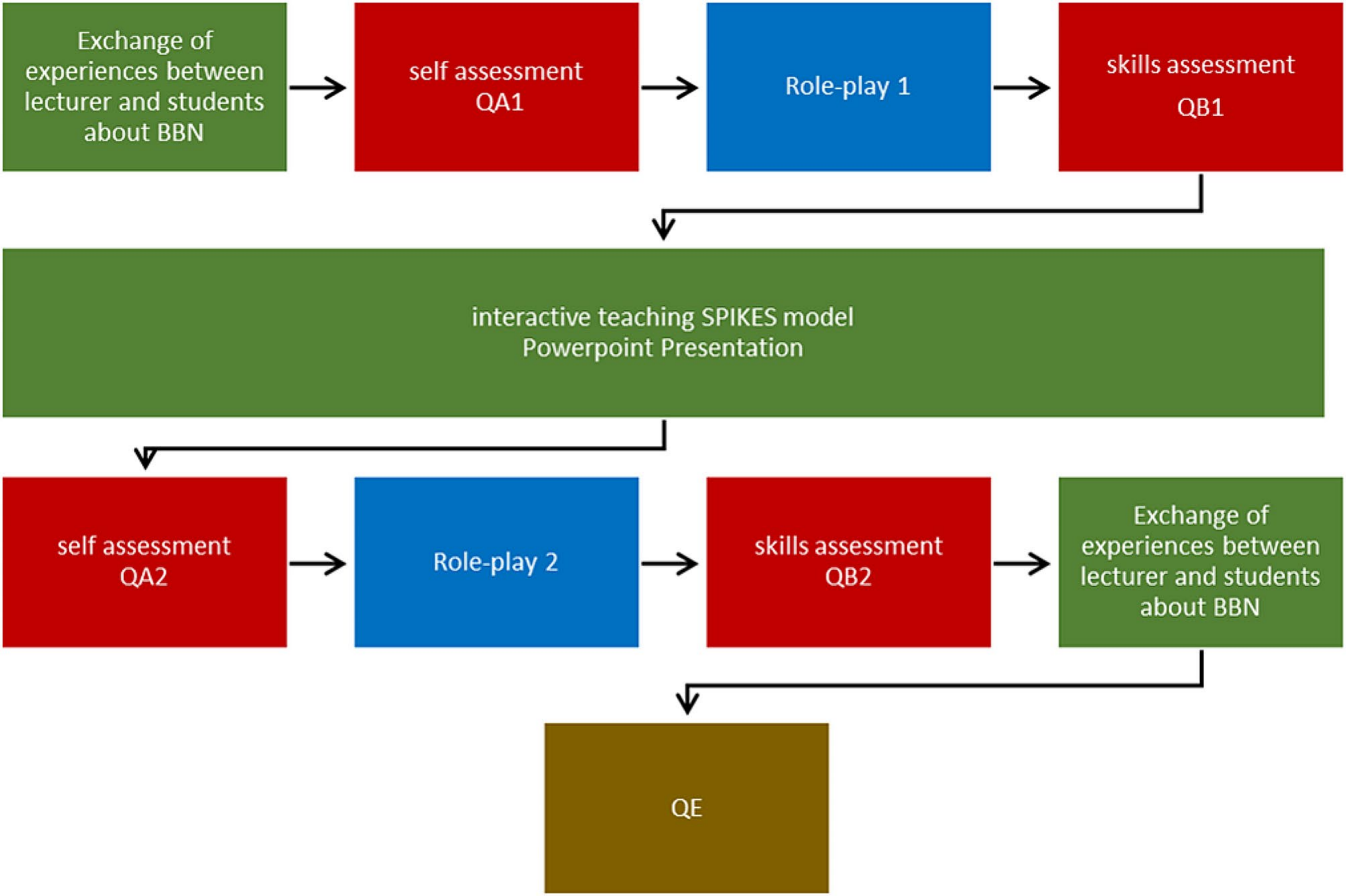
Sequence of the BBN seminar. (BBN = Breaking Bad News; QA1 = questionnaire A to evaluate the students’ self-assessment before lesson; QB1 = questionnaire B to evaluate the students’ communication skills during the role-play before lesson; SPIKES = Setting, Perception, Invitation, Knowledge, Emotions, and Strategy and Summary; QA2 = questionnaire A to evaluate the students’ self-assessment after lesson; QB2 = questionnaire B to evaluate the students’ communication skills during the role-play after lesson QE = questionnaire for seminar evaluation)

The training model proposed in this publication consisted of two sections: theoretical training and practical training including role-plays. To assess the development of the students’ self-assessment in terms of self-confidence, importance, and their own interest in BBN before and after the seminar, one questionnaire each (QA1/QA2) was distributed and collected immediately at the beginning of the seminar and immediately after the interactive teaching (Fig. 1). To evaluate the impact of the teaching in the development of the students’ communication skills one questionnaire each (QB1/QB2) was distributed before the role-plays and collected after (Fig. 1).

The performers received a brief written introduction to the fictitious role-play characters based on real cases which were created by the authors (Supplement 1). In the role of a junior doctor, the students’ task was to explain to a patient that she has a histologically confirmed diagnosis of breast cancer (BBN), but the recommendation for further therapy has not yet been determined because of other pending results. The role of the patient was individualized: the student who took on the role of the patient was instructed to react based on her individual personal background. In the second role-play the patient had different characteristics. The observers were seated nearby the roleplay to observe. They obtained the QB sheet at the beginning of the role-play to be able to rate the items. For the role-play, students were organized into groups with a minimum of 3 students (one physician, one patient, one observer). A timeframe was given and the role-play itself should not exceed 15 min. The participants were asked to voluntarily play the role of the patient or the physician. If necessary, a lot was drawn. A debriefing was conducted after the role-plays respectively. During the debriefing, the participants were encouraged to describe their feelings about breaking bad news, in case of being the physician, and how they felt about receiving bad news, in case of being the patient, during the role play. The debriefing was held as a 360°feedback involving all students and the lecturer. This allowed a formative self and peer- assessment cycle to continuously improve the student’s attainment and the lecturer’s didactic strategy.

Thus, the first part of the seminar consisted of an interactive introduction related to lecturer and student experiences with BBN (a brief definition of bad news, examples of bad news communications not only in oncological settings), filling out the questionaries’ and performing the first role-play. The mean duration was 30 min.

**Fig. 2:**
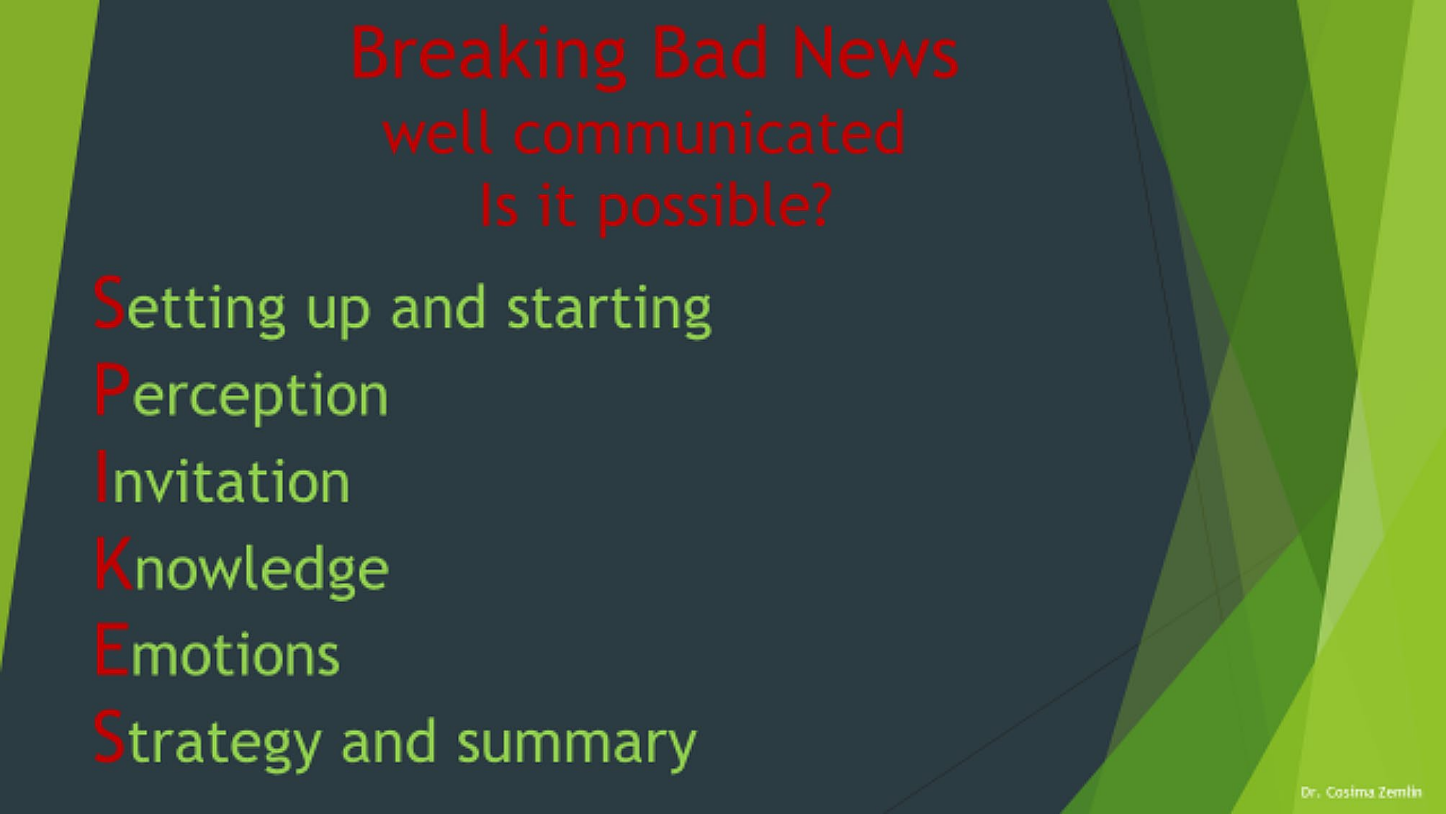
A slide from the interactive lecture on the SPIKES-protocol for breaking bad news

The second part consisted of the theoretical training with an average duration of 30 min (Fig. 2). It included a PowerPoint™ presentation (Supplement 3) based on the SPIKES protocol. Every single step of the SPIKES protocol for “breaking bad news” was taught and discussed. This was followed by a brief introduction to gyneco-oncology and the needs of the patients based on the lecturer’s experiences.

The last part of the seminar consisted of the second role-play, filling out the questionnaires, a final debriefing in the sense of a formative self-and peer assessment. Students had time to ask questions and get answers. The 360°feedback included the evaluation questionnaire about the whole seminar (QE) and an encouragement of the students to give feedback about the training to the lecturer directly. The mean duration was 30 min.

All questionnaires were anonymous. Questionnaire QA and QB, written introduction to the fictitious role-play and lecture slides are provided in Supplementary Data (Supplement 1–3).

### Data analysis

Categorical variables are represented as absolute frequencies or both absolute and relative frequencies. Ordinal variables are illustrated by displaying the median with the interquartile range (IQR) represented by [1st quartil– 3rd quartil]. Since the questionnaires were submitted anonymously and there is no way to link the pre- and post-questionnaires, we used statistical tests for independent samples. So, ordinal variables were compared using the Mann-Whitney U test and categorical variables were compared by the Fisher-Freeman-Halton exact test. Any *p* values are two-sided.

Statistical analyses were performed using SPSS 29.0 (IBM, Armonk, USA) software. A *p*-value of less than 0.05 was considered significant for all tests.

## Results

158 students attended the BBN Seminar in the summer semester 2023. Classes were bigger in the beginning of the semester. Except for the last class, it was always possible to provide 4 doctors and 4 patients respectively for all role-plays. In the last seminar there were 3 doctor- patient- observer groups (Table 1).

**Table 1:** Students attending the BBN (breaking bad news)- seminar and distribution in physicians, patients, and observers during the role-play

	group size	physicians	patients	observers
Students total	158	31	31	96
Class 1	28	4	4	20
Class 2	24	4	4	16
Class 3	21	4	4	13
Class 4	23	4	4	15
Class 5	16	4	4	8
Class 6	16	4	4	8
Class 7	20	4	4	12
Class 8	10	3	3	4

The results of the QA1/2 and QB1/2 questionnaires are shown in Tables 2 and 3.

**Table 2:** Evaluation of the 11-point Likert scale of the QA- (students’ self-assessment) and QB (peer assessment) questionnaires and presentation of the changes in the ratings over the course of the test (QA/B 1 to 2) using the Mann-Whitney U test (*p* < 0.05 significant, *p* < 0.001 highly significant). (n = number of responses, IQR = interquartile range)

	total Q1 (n)	median (%)	IQR (%)		total Q2 (n)	median (%)	IQR (%)	*p*
QA								
1. self-confidence in BBN	158	40	[30–60]		155	70	[50–80]	< 0.001
2. interest in gyneco- or oncology	158	50	[20–80]		155	50	[30–80]	0.287
3. understanding gyneco-oncology	158	30	[20–50]		155	50	[40–70]	< 0.001
4. understanding for CiO	157	60	[40–70]		155	80	[65–90]	< 0.001
5. self-confidence for CiO	158	40	[20–50]		155	60	[50–70]	< 0.001
6. personal importance of CiO	158	90	[80–100]		155	100	[80–100]	0.110
7. own relevance of the seminar	157	80	[60–90]		154	90	[70–100]	0.011
QB								
1. conversation at eye level	157	90	[80–100]		154	90	[70–100]	0.541
2. times, doctor made eye contact	157	90	[80–100]		154	90	[70–100]	0.735
3. doctor showing empathy	158	80	[70–100]		154	80	[70–98]	0.664
4. understandable language	156	80	[63–90]		154	80	[70–90]	0.273
5. time to think for the patient	158	70	[50–90]		154	80	[70–90]	0.057
6. time to ask questions	158	90	[60–100]		154	80	[70–100]	0.983
7. doctor given a summary	153	70	[50–90]		154	80	[60–100]	< 0.001

**Table 3:** Evaluation of the 2-point Likert scale of the QA- (students’ self-assessment) and QB (peer assessment) questionnaires and presentation of the changes in the ratings over the course of the test (QA/B 1 to 2) using the Fisher-Freeman-Halton exact test (*p* < 0.05 significant, *p* < 0.001 highly significant). (n = number of responses, IQR = interquartile range)

	Total Q1 (n)	yes (n)	no (n)	indifferent (n)	yes (%)	Total Q2 (n)	yes (n)	no (n)	indifferent (n)	yes (%)	*p*
QA											
8. use of role-plays in the studies	152	103	46	3	68	154	111	41	2	72	0.676
9. content of seminar being part of lectures	151	116	32	3	77	154	117	36	1	76	0.798
10. seminar be offered as an elective	152	133	15	4	88	154	138	15	1	90	0.711
QB											
8. doctor initially asking an open question	156	61	94	1	39	150	123	27	0	82	< 0.001
9. offering to bring an accompanying person	155	23	130	2	15	150	80	66	4	53	< 0.001
10. providing helpful material	156	15	139	2	10	149	99	49	1	66	< 0.001
11. reassurance of patients’ understanding	152	40	111	1	26	144	67	72	5	47	< 0.001
12. drawing or writing for the patient	157	5	152	0	3	149	26	121	0	18	< 0.001
13. aim is to cure the patient	154	132	16	6	86	149	124	20	5	83	0.761
14. ideas to gain back self-control	155	20	133	2	13	146	85	59	2	58	< 0.001
15. clear and honest communication	156	128	23	5	82	148	122	19	7	82	0.737
16. Was to doctor calm?	156	149	7	0	96	150	133	14	3	89	0.039
17. explanation of the patient’s “rights”	153	22	130	1	14	145	42	103	0	29	0.003

The items for students’ self-assessment in questionnaire QA1/2 show an improvement in all areas, all of which are significant except for item 2 (“interest in gyneco- or oncology”) and 6 (“personal importance of communication in oncology”). For question 6, the median value of the rating increased from 90 to 100%, for question 2 the rating is similar from 50 to 50% (Table 2).

In the initial survey, 68% of the students wished for role-plays in their studies, 77% wanted the topic to be part of lectures and 88% wanted them to be part of further seminars. In the second survey after the interactive teaching, 72% of the students wanted role-plays in their studies, 76% wanted the topic as part of lectures and 90% of other seminars (Table 3).

The items used to evaluate the students’ skills using the QB1/2 questionnaire (peer-assessment) showed no significant differences on the 11-point Likert scale (Table 2) except for the item 7 (“Did the doctor summarize the essentials at the end of the conversation?”). This item showed a highly significant improvement. The ratings in the second part of the QB1/2 questionnaire using a 2-point Likert scale (yes/no), showed significant improvements in questions 8–12, 14, and 17. Questions 13 and 15 showed no significant differences. Item 16 decreased significantly from 96 to 89% (Table 3).

The students rated the seminar on a 5-point Likert scale (1="strongly disagree” to 5="absolutely agree”) with 10 items (QE) in the sense of a 360°feedback. Almost all points were rated with a median score of 5. The questions “I learn a lot in the event” and “The event promotes my interest in the subject” scored a median of 4, respectively (Table 4).

**Table 4:** Evaluation of the 5-point Likert scale of the QE- (Questionnaire for seminar evaluation) using the Fisher-Freeman-Halton exact test. The evaluation was carried out using grades 1 to 5. (*p* < 0.05 significant, n = number of responses, IQR = interquartile range)

Questions	total QE (n)	Grades: 1 strongly disagree to 5 absolutely agree					median	IQR
		1	2	3	4	5		
1. The lecturer can make complicated things understandable	146	1	1	5	38	101	5	[4–5]
2. Own contributions, questions and active participation are encouraged	147	1	1	6	14	125	5	[5–5]
3. The students receive helpful feedback on their questions	147	0	2	14	27	104	5	[4–5]
4. Theory and practice are well coordinated	146	1	1	8	45	91	5	[4–5]
5. The lecturer is open	146	0	1	5	23	117	5	[5–5]
6. The lecturer represents the subject with commitment	147	0	0	4	12	131	5	[5–5]
7. I learn a lot in the event	147	1	7	20	58	61	4	[4–5]
8. The event promotes my interest in the subject	147	8	13	32	46	48	4	[3–5]
9. The technical/content quality of presentations is high	144	0	4	10	39	91	5	[4–5]
10. What grade would you give the lecturer overall?	142	0	1	13	37	91	5	[4–5]

## Discussion

Despite of numerous approaches for communication skill training (CST) existing for medical students, especially for BBN some questions remain unanswered: When to start training, which framework and setting is effective and at what costs?

We postulate that it is a good time to start CST for medical students even before entering the clinical phase of their studies. According to the German curriculum, students had gained some clinical experience in their nursing internships before the end of their 2nd year of studies. Additionally, students also experience in the private sector of how bad news are broken by physicians in everyday medical practice. In our seminar, most of the students talked about these experiences and the desire to do better. All students had an opinion on the subject, although they classified themselves as rather inexperienced. Using a formative self- and peer assessment with 360°feedback during the seminar, we could show that both the students’ self-confidence and skills improved significantly [30]. In Germany, longitudinal curricula are now recommended, which should offer the topic of BBN several times during medical studies using different training settings (Masterplan; NKLM 2.0 [23]). Thus, teaching communication skills especially BBN, is a mandatory part of medical education. Therefore, it must be integrated in most subspecialties, should not be taught separately [31] and validated teaching concepts for BBN seminars are urgently needed. In addition, despite the implementation of CST into medical curricula, it is not guaranteed that the skills are learned and acquired in a sustained way, since different interest of the students influence the acceptance and application to offered seminars [32]. The acceptance of such seminars could be demonstrated in those studies on CST recruiting volunteer participants [32]. In contrast to the latter study, our seminar was compulsory, ruling out a selection bias as all students were required to attend the seminar regardless of their interest in CST and BBN. Interestingly, students’ self-confidence and skills improved significantly in our study.

Depending on the teaching strategy, student’s assessments of their peer’s communication skills may significantly differ from the judgement of real patients [12]. Thus, it is a limitation of our study that the feedback of students to their peers may not reflect the true needs of the patients. The assessments of communication skills were already very good in the first questionnaire, so it was hardly possible to improve them in some cases. Moreover, although this study reports a relatively large number of participants in comparison to other reports, our group of students might not be representative for others. However, the student’s performance in BBN was also assessed by an experienced lecturer.

The use of anonymous questionnaires makes it impossible to link individual pre and post answers of the participants. Thus, we cannot identify individual or group specific predictors that would allow more individualized teaching strategies. Future studies should consider using personal identifiers to overcome this limitation.

We conclude that it is important to evaluate different settings for communication skill training at various time points within medical studies. Despite our rather simple setting using peer role play actors, the students’ interest in the topic increased during the seminars. Most students asked for further opportunities to learn BBN and communication skills in other settings, e.g., lectures and other seminars. Although the seminar was held as a compulsory course in the afternoon after a full day of studying, the students rated the course very well. They even stated that their interest in the topic had increased together with their communication skills.

We chose the SPIKES protocol as it is well documented that it serves the most important needs of the patients and gives the doctor an easily applicable guide for a successful conversation [1]. But further studies are needed to evaluate the effects of other programs.

The students stated that the setting with repeated role play was very helpful. In particular, the students reported to benefit very much from putting themselves into the patient’s role. The rating was even better than in other programs with actors [28].

Our teaching model is very effective, very well rated, and significantly increased communication skills even in undergraduate medical students. Since professional actors are unnecessary, our teaching strategy using peer role plays and assessment is inexpensive and easy to establish longitudinally during medical studies.

Communication and delivering bad news are important skills that can be learned, like any other skill in medicine [11]. During the second round of role plays, the “physicians” were rated to appear less “calm” in comparision to the first role play. This could be due to an increased awareness of the students for the importance of BBN and therefore increased arousal.

The fear of BBN can turn into satisfaction if it succeeds [11]. There is compelling evidence that good communication skills are important for patients as well as for doctors. Training for breaking bad news can sharpen the perception of students and doctors for the aspects that are important in physician-patient communication [17]. The awareness and skills of the participants can be increased by BBN seminars with formative self-and peer assessment and due to the direct exchange of experience, it can also be instructive even for the experienced teachers to hold BBN seminars using 360°feedback [30].

## Conclusion

We postulate that, first, it is possible and recommended to start communication skill training in the pre-clinical phase of medical studies. Role-plays can be conducted by students themselves, thus these seminars require little staffing and are cost-effective. Second, even more important, students experience increased self-awareness when participating in the role-play and by serving as observers. Communication skills should be taught repeatedly during seminars of multiple subspecialties and should be an integral and compulsory part of medical curricula using formative assessments. Despite the limitations described above, our study adds to the knowledge of BBN among medical students and generates insights that can be used in future research and interventions to improve medical students’ skills for BBN. Successor studies should aim to examine the effectiveness of various teaching programs and the impact on the real-life doctor-patient conversation during clinical routine.

### Electronic supplementary material

Below is the link to the electronic supplementary material.

Supplementary Material 1

Supplementary Material 2

## Data Availability

The datasets supporting the conclusions of this article are included within the article or are available from the corresponding author (Sybelle.Goedicke-Fritz@uks.eu) upon reasonable request.

## Abbreviations

BBN: Breaking Bad News
CiO: Communication in oncology
CST: Communication skill training
EKM: **E**inführung in die **k**linische **M**edizin “Introduction to clinical medicine ”
QA: Questionnaire A to evaluate the students’ self-assessment
QB: Questionnaire B to evaluate the students’ communication skills during the role-play
QE: Questionnaire for seminar evaluation
SPIKE: Setting, Perception, Invitation, Knowledge, Emotions and, Strategy and Summary
