# Supplementary material for: Teaching breaking bad news in a gyneco-oncological setting: a feasibility study implementing the SPIKES framework for undergraduate medical students
Source: BMC Med Educ. 2024 Feb 12;24:134. doi: 10.1186/s12909-024-05096-9 (PMC10863240; doi:10.1186/s12909-024-05096-9)
Supplement: Supplementary file 1 — Supplementary Material 1 [file 12909_2024_5096_MOESM1_ESM.pdf]

Breaking bad news

EKM 2023

Dr. Cosima Zemlin

Breaking Bad News
well communicated

Is it possible?

Dr. Cosima Zemlin

Breaking Bad News
well communicated
Is it possible?

Setting up and starting

Perception

Invitation

Knowledge

Emotions

Strategy and summary

Setting up and starting

- Am I prepared? (review medical findings)
- Do I have everything I need?
  (Documents (Copies for the patient),
  Flyer (Support groups, Counseling centre, Handout))
- Take a deep breath

- Setting:
- undisturbed?
- at eye level?
- accompanying person desired?

suitable setting?

Perception

- Look at the patient: who is sitting in front of me?
- Introductory open question
- What does the patient know already?
- Empathy, put yourself in the patients place
- Allow breaks
- Listen carefully

suitable
setting?

Invitation

It‘s about the patient

- Look at the patient
- Ask if there are questions (repetitive)
- Listen carefully
- Right not to know

suitable setting?

Knowledge

Convey information

- Look at the patient
- Radiate calmness
- Easy wording
- Make medical findings clear, use words like cancer, death, fear
- Paint or write things down
- Allow breaks
- Hand out information material, also inform about patients rights (CAVE too much for once)

suitable setting?

Emotions

Gefühl ist erlaubt

- Look at the patient
- Radiate calmness
- Show empathy
- Allow breaks
- Speak about emotions
- How do you feel now?
- What do you want / need? (patients decision!!!)
- Convey hope!!!
- Take away fears!!!

suitable setting?

Strategy and summary

- Look at the patient
- Radiate calmness
- Easy wording
- Hand out information material
- Summarize the essentials and repeat them again
- Room for questions (advice, recommend to note questions for the next meeting)
- In an adjuvant situation, emphasize HEALING

suitable setting?

Breaking Bad News
communicated well

Setting up and starting.

Perception

Invitation

Knowledge

Emotions

Strategy and summary

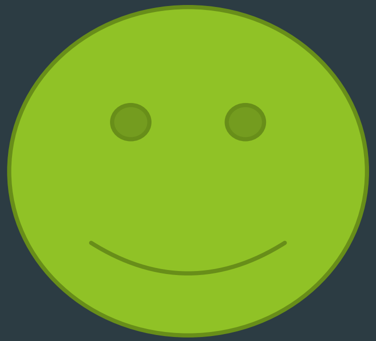

Dr. Cosima Zemlin

Gyneco- Oncology?

1. Question : Curable vs not curable?
2. Question : Chemotherapy?
3. Question : What can I do?
4. Question : Am I guilty?

1. Curable vs not curable
2. Chemotherapy?

- Type of cancer (histology, e.g. invasive breast cancer, NST, G2)

- Stage: TNM =
  T = size, N = lymph node involvement, M = metastasis

- Additional information to estimate the risk of recurrence/metastasis
- (e.g. hormone receptor status)

- Age and health status of the patient

- Puzzle metaphor : “We need all the puzzle pieces to have a clear picture and know what is best for you!”

Therapy for breast cancer?

1. Operation
2. Radiation

---

3. Anti-hormonal therapy
4. Chemotherapy
5. Antibodies

What can I do?

1. Sports
2. Healthy diet
3. Psycho-oncology
4. Folder, to write down questions
5. Advice center, support group, health insurance

Thank you and good luck!

☺

Thank you and good luck!

☺

PS.: Humor helps too!!!
