# Supplementary material for: Teaching breaking bad news in a gyneco-oncological setting: a feasibility study implementing the SPIKES framework for undergraduate medical students
Source: BMC Med Educ. 2024 Feb 12;24:134. doi: 10.1186/s12909-024-05096-9 (PMC10863240; doi:10.1186/s12909-024-05096-9)
Supplement: Supplementary file 2 — Supplementary Material 2 [file 12909_2024_5096_MOESM2_ESM.pdf]

Supplement 1: Role-Play

1

Role of the doctor:

Young assistant doctor (1/2 year after exam), all colleagues are in the operating room.

A patient has been waiting for 2 hours and should be informed about the histological findings (see below). A new appointment (approx. in a week) should also be arranged to plan the further procedure once all histological results are available. Thus, the exact planning of the further procedures will be next week.
The patient has an initial diagnosis of breast cancer: T = tumor size in the right breast according to ultrasound is 0.9 cm, L = lymph nodes in the axilla seem unaffected = L0. Further results such as receptors, which are important for further therapy planning, are not yet available. The patient probably "only" needs surgery of the breast and axilla (sentinel lymph node), followed by radiation and anti-hormonal therapy with one tablet daily for 5 years. This is unclear as long as the receptors are missing. In this case, chances of healing would be around 98%.

1

Role of the patient:

51 years old, married, 2 children 14 and 17 years old.

Her husband is at work, the patient does not have anyone accompanying her today.

She had felt a lump in her breast 3 weeks ago and went to the gynecologist 2 weeks ago, who referred her to the clinic for a punch biopsy (5 days ago).
There is no history of cancer in the family. However, last year a neighbor died of breast cancer.

The patient suspects that it is cancer, but does not want to admit it (yet).

The patient has been waiting for 2 hours. She should be informed about the histological findings today.

2

Role of the doctor:

Young assistant doctor (1/2 year after exam), all colleagues are in the operating room.

A patient has been waiting for 2 hours and should be informed about the histological findings (see below). A new appointment (approx. in a week) should also be arranged to plan the further procedure once all histological results are available. Thus, the exact planning of the further procedures will be next week.
The patient has an initial diagnosis of breast cancer: T = tumor size in the right breast according to ultrasound is 0.9 cm, L = lymph nodes in the axilla seem unaffected = L0. Further results such as receptors, which are important for further therapy planning, are not yet available. The patient probably “only” needs surgery of the breast and axilla (sentinel lymph node), followed by radiation and anti-hormonal therapy with one tablet daily for 5 years. This is unclear as long as the receptors are missing. In this case, chances of healing would be around 98%.

If there is a history of breast cancer in the family, it could be a so-called triple negative breast cancer. In this case the patient would need chemotherapy and antibody therapy, possibly even before the surgery, but, that is not yet clear. A genetic test would be recommended if, for example, there are already two cases of breast cancer in the family and the patient is the third to suffer from it.

2

Role of the patient:

51 years old, mot married, no children

The patient does not have a companion with her, but the sister who had breast cancer two years ago and needed chemotherapy “and the whole program” could come with her to the next appointment. The sister is now doing well. The doctors say she is cured. But her mother died of breast cancer 10 years ago.

She had felt a lump in her breast 3 weeks ago and went to the gynecologist 2 weeks ago, who referred her to the clinic for a punch biopsy (5 days ago).

The patient suspects that it is cancer, but does not want to admit it (yet).

The patient has been waiting for 2 hours. She should be informed about the histological findings today.

Supplement 2: Questionnaire

Questionnaire self-assessment (QA):

1. How much do I dare to conduct an explanatory talk about a cancer diagnosis in a patient at this time (regardless of professional competence)?

0%	10%	20%	30%	40%	50%	60%	70%	80%	90%	100%

2. How interested are you in the field of gynecology or oncology?

0%	10%	20%	30%	40%	50%	60%	70%	80%	90%	100%

3. How do you rate your understanding of gyneco- oncology?

0%	10%	20%	30%	40%	50%	60%	70%	80%	90%	100%

4. How do you rate your understanding of communication in oncology?

0%	10%	20%	30%	40%	50%	60%	70%	80%	90%	100%

5. How do you rate your confidence for communication in oncology?

0%	10%	20%	30%	40%	50%	60%	70%	80%	90%	100%

6. How do you rate the importance of communication in oncology for you personally?

0%	10%	20%	30%	40%	50%	60%	70%	80%	90%	100%

7. How do you rate the relevance of the seminar on communication in oncology for your future medical profession?

0%	10%	20%	30%	40%	50%	60%	70%	80%	90%	100%

	**yes**	**no**
8. Should role-plays be offered more often during the studies?	O	O
9. Should content of the seminar be part of a lecture?	O	O
10. Should the seminar be offered as an elective to have more time?	O	O

Questionnaire communication skills (QB):

1. Was the conversation at eye level - in the truest sense?

0%	10%	20%	30%	40%	50%	60%	70%	80%	90%	100%

2. Percentage of times the doctor made eye contact.

0%	10%	20%	30%	40%	50%	60%	70%	80%	90%	100%

3. Does the doctor show empathy?

0%	10%	20%	30%	40%	50%	60%	70%	80%	90%	100%

4. Has the "doctor" explained enough technical terms or explained them in layman's terms?

0%	10%	20%	30%	40%	50%	60%	70%	80%	90%	100%

5. Was there enough time for the patient to think during the conversation?

0%	10%	20%	30%	40%	50%	60%	70%	80%	90%	100%

6. Was there enough time during the dialog for the patient to ask questions?

0%	10%	20%	30%	40%	50%	60%	70%	80%	90%	100%

7. Did the doctor summarize the essentials at the end of the conversation?

0%	10%	20%	30%	40%	50%	60%	70%	80%	90%	100%

Please turn, continue on the back

		yes	no
8.	Did the doctor initially ask an open question?	O	O
9.	Has the doctor offered the patient to bring along an accompanying person?	O	O
10.	Did the doctor provide information material/flyers? E.g. for self-help groups, advice centers, second opinion?	O	O
11.	4. Did the doctor ask what the patient understood?	O	O
12.	Did the doctor draw/write something for the patient?	O	O
13.	At the end of the conversation, did the doctor make it clear that the aim is to cure the patient (adjuvant situation)?	O	O
14.	Did the doctor give the patient something to do to get back her/his control? (What can you do for yourself? E.g., sport, nutrition, writing diary, create folders, write down questions)	O	O
15.	Does the doctor communicate clearly, without leaving much room for interpretation, especially when it comes to the “bad news”?	O	O
16.	Does the doctor communicate calmly and reassuringly - including body language?	O	O
17.	Did the doctor explain the patient's "rights"? (What aids, courses, travel expenses, etc. could be covered by health insurance)?	O	O
